# Examining the Impact of Social Connectedness on Depression and Suicide Ideation Among Black Youth Experiencing Discrimination: A Path Analysis

**DOI:** 10.1007/s40615-025-02414-9

**Published:** 2025-04-16

**Authors:** Brianna L. Smith, Donte T. Boyd, Camille R. Quinn

**Affiliations:** 1 College of Social Work, The Ohio State University, Columbus, OH USA; 2 School of Social Work, University of Michigan, Ann Arbor, MI USA

**Keywords:** Black youth, Discrimination, Social connectedness, Depression, Suicide ideation

## Abstract

Research has found that experiences of racial discrimination among Black youth contribute to adverse behavioral health outcomes, including depression and suicide ideation. This study is guided by social support theory and the stress buffering model to further understand how social connectedness can influence the effect of everyday discrimination on depression symptoms and suicide ideation. Using survey data from a sample of 362 Black youth (18 to 24 years of age) in the Midwest, the current study conducted path analyses to examine the direct and indirect effects of everyday discrimination on depressive symptoms and suicide ideation, mediated by social connectedness. The average age of the sample was 21 (*SD* = 1.96), and the majority of the sample self-identified as female (70%). Fifty-one percent of the sample reported suicide ideation. Study results found that everyday discrimination was directly associated with an increase in depression symptoms and suicide ideation. Results also indicated that social connectedness explained lower suicide ideation and depression symptoms through everyday discrimination. Implications for behavioral health practices addressing race-based psychosocial stress, and directions for future research are discussed.

## Introduction

### Racial Discrimination, Health, and Well-Being

Racial discrimination is an ongoing public health crisis that fuels health disparities among Black Americans in the United States (US) [1]. Black youth disproportionately experience racial discrimination within their lifetime. As early as 10 years of age, Black children report experiences of racism and discrimination [2]. Research examining the prevalence of perceived discrimination among children aged 10–11 years found that Black children experience the highest incidence of racial and ethnic discrimination in the US [3]. Throughout Black youth’s development, evidence suggests that experiences of racial discrimination significantly increase [2, 4]. Within adolescence and young adulthood, Black youth are subjected to greater, more frequent racial discrimination experiences through broadening social systems. Broadening social systems subjecting Black youth to heightened exposure to interpersonal and structural racial discrimination include secondary or higher education, occupational workplaces, healthcare, and law enforcement [4]. The disproportionate racial discrimination experiences among Black youth in the US are a result of chronic, prolonged exposure to bigoted practices and socialization within historically oppressive systems and social structures. Characterized by the oppressive social construction of Black Americans embedded within the sociopolitical history of the US, these bigoted practices and socialization encompass discriminatory policies, regulations, behaviors, or attitudes inducing race-based experiences of social inequality [2]. Racial discrimination is defined as the unequal, differential treatment of a person or social group based on race/ethnicity [5]. Behaviors encompassing racial discrimination include distinction, exclusion, restriction, or preferential treatment, which contribute to political, economic, and social inequality that exacerbate disparities in adverse behavioral health outcomes [6, 7]. Experiences of racial discrimination directly and/or indirectly impact health outcomes, as racial discrimination may be experienced directly, indirectly, or vicariously, contributing to phenomena known as racial stress [8, 9]. Experiences of race-based stressors, such as racial discrimination, invoke a psychological, behavioral, or physiological response that negatively impacts an individual’s health and well-being [6, 10].

### Racial Discrimination and Depression Symptoms Among Black Youth

Black youth’s experiences of psychosocial stressors, including racial discrimination, place them at a greater risk of poor mental health outcomes [11, 12]. Research suggests that experiences of racial discrimination evoke psychological and physiological stress responses that can result in the development of behavioral health disorders characterized by experiences of helplessness, negative self-perception, loss of perceived self-control, and maladaptive coping mechanisms [13–15]. Investigations of the relationship between racial discrimination and behavioral health disorders suggest that racial discrimination is associated with depression among Black youth [16, 17]. A longitudinal research study using data from the Protecting Strong African American Families (ProSAAF) project examined the association between racial discrimination and depression among a sample of 346 Black youth who were between 9 and 14 years of age and resided in rural southern communities in the US. The results of the study indicated that greater reports of racial discrimination are associated with greater symptoms of depression, and that racial discrimination is predictive of depressive symptoms [18].

### Racial Discrimination and Suicidality Among Black Youth

Suicide is the second leading cause of death in the US for youth ages 15 to 24 [19]. Research by Twenge et al. shows an alarming increase in nonfatal suicidal behaviors among youth, revealing a 46% rise for 18 to 19 years old, a 68% increase for individuals ages 20 to 21, a 55% escalation for those in the 22- to 23-year-old age group, and a 29% uptick for 24 to 25 years old [20]. Racial disparities in suicidality are particularly concerning, for evidence demonstrates that Black youth are experiencing a rise in suicide rates in the US [21]. Trends among Black youth from 2001 to 2017 suggest a major rise in suicidal ideation, plans, and attempts necessitating medical treatment [22, 23]. Between 1999 and 2019, suicidal thoughts and behaviors among Black youth ages 10 to 14 increased at a rate of 131.5% [24–26]. From 2003 to 2017, Black youth ages 15 to 17 had the largest rise in yearly percentage changes in suicides, from 4.9 to 6.6% [22, 23].

The alarming rates of suicide among Black youth are a pressing issue that requires attention and action [19]. Recent studies emphasize the need for additional investigation into the impact of racial discrimination on mental health outcomes. Findings suggest that racial discrimination serves as a substantial social risk factor associated with suicidality and capability for suicide among racial minority populations in the US [27–29]. Evidence suggests that youth who experience exclusion and social disparagement are vulnerable to suicidal behaviors [30], placing Black youth subjected to racial discrimination at greater risk for suicidality. Supporting the relationship between social disparagement and greater suicidality, research examining the relationship between racial discrimination and suicidality among Black youth found that perceived discrimination was associated with greater odds of suicide ideation [31–33].

### Social Connectedness and Mental Health Outcomes

Research across health disciplines has found that social connectedness is a determinant of health that promotes positive mental health outcomes [34]. Social connectedness is conceptualized as an interrelated, higher-order construct of social support, as it focuses on the significance of interpersonal closeness within collective relationships that satisfy the social, emotional, and psychological needs of an individual [35–37]. Evolving research provides support for the relationship between social connectedness and mental well-being, for recent literature has found that community connectedness is associated with reduced symptoms of depression and suicide ideation [38, 39]. In a sample of 212 Black high school students, a study aiming to assess risk and protective factors of suicidality among Black youth examined the effects of family support, peer support, and community connectedness on suicidality and depression. Researchers found that family and peer support and community connectedness were protective factors for suicidality. The results suggest that community connectedness had both direct and indirect effects on decreased suicidality, for community connectedness moderated the relationship between depression and suicidality [40], providing support for the significance of social connectedness in reducing suicide ideation among Black youth.

Research has also found that community connectedness improves an individual’s ability to adaptively respond to adversity through the provision of support that serves as a protective factor against poor behavioral health outcomes [41, 42]. A study examining the relationship between social connectedness, racial discrimination, and posttraumatic stress found that social connectedness within ethnic communities significantly moderated the relationship between racial discrimination and posttraumatic stress among Chinese international students in the US. Although these constructs were not examined among Black youth, these findings provide support for the significance of social connectedness in mitigating the impact of racial discrimination on mental health outcomes among racial and ethnic minorities [43].

### Guiding Theoretical Frameworks

Among Black youth experiencing a developmental transition of independence, social connectedness is of particular importance in cultivating social relationships that foster social support. Informed by social support theory positing that positive interpersonal relationships promote health and well-being within experiences of adversity through resilience processes known as stress buffering, this study examines the role of social connectedness as a mediator among racial discrimination, depressive symptoms, and suicide ideation [44]. Social support theory examines the impact of social relationships on individual well-being, coping mechanisms, and health outcomes. The theory suggests that the presence of supportive relationships can buffer individuals from the negative effects of stress and adversity, thus promoting better physical and mental health. It also suggests that perceived social support may protect individuals against stressors by influencing their interpretation of adverse situations (i.e., helping them to adopt a more positive perspective on a challenging circumstance) [45]. Thus, Black youth may effectively regulate their responses to these stressors if they can assess the availability and quality of the various forms of support available to them [46, 47]. Previous literature has illustrated that Black youth who perceive greater availability and quality of social support are less prone to experiencing psychological distress [47–49]. Further, research has shown that strong social support, including close relationships and consistent communication with family and friends, significantly contributes to increasing life satisfaction and reducing psychological distress [47, 50].

The stress buffering model suggests that social support can either mitigate or moderate the adverse effects of stress on health [47, 51]. Perceived social support could serve as a shield against the harmful impacts associated with life stressors and decrease the likelihood of negative health outcomes [47, 51]. Research in this area suggests that these relationships may arm Black youth with additional coping mechanisms and foster resilience [47, 52]. These discoveries underscore the necessity for further investigation into the role of social connectedness in the relationship between racial discrimination and suicide ideation among Black youth.

### The Current Study

In developing solutions that mitigate the impact of racial discrimination on depression and suicide ideation among Black youth, it is essential that researchers identify protective factors that positively influence behavioral health outcomes. Despite existing literature examining the impacts of social connectedness on the behavioral health outcomes of racial/ethnic minority communities reporting experiences of racial discrimination [43, 53], there is limited research exclusively examining the impacts of social connectedness on the behavioral health of Black youth [39, 54, 55]. Across empirical evidence providing support for the significance of social connectedness on the psychosocial well-being of Black youth, there is particularly a lack of research examining the impact of social connectedness on the associative relationship between racial discrimination and adverse behavioral health outcomes including depression and suicide ideation.

Expanding research examining the significance of social connectedness on the behavioral health of Black youth is critical, for social connectedness builds resilience in combatting psychosocial stress through improved psychological and emotional functioning [56]. This protective mechanism is of particular importance among Black youth experiencing race-based psychosocial stress during a developmental period in which social relationships and behavioral health needs evolve throughout an individual’s emergence into adulthood [57]. The purpose of the present study is to expand existing research by examining social connectedness as a protective factor in mitigating the impacts of racial discrimination on depression and suicide ideation among Black youth and by providing evidence supporting the underlying mechanisms in which social connectedness mitigates the impact of racial discrimination on the behavioral health of Black youth.

According to the statistical definition provided by the United Nations, “youth” refers to individuals aged 15 to 24 years. This term encompasses a developmental period that marks the transition from childhood to adulthood, including both adolescence and young adulthood [58]. In this study, the term “Black youth” is used to describe the sample of participants we are examining. Guided by social support theory and the stress buffering model, we propose the following research questions: first, does social connectedness among Black youth mediate the relationship between discrimination and depression symptoms? And second, does social connectedness among Black youth mediate the relationship between discrimination and suicide ideation? In examining the proposed research questions, we assessed the following hypotheses:

1. Discrimination will be positively associated with suicide ideation and depression symptoms.
2. Social connectedness will mediate the relationship between discrimination and depression symptoms.
3. Social connectedness will mediate the relationship between discrimination and suicide ideation.

## Methods

### Participants and Procedures

This study used Qualtrics panels to collect a sample of Black youth ages 18 to 24 from the Midwestern United States (*N* = 362) to examine their experiences with mental health, racism, and sexual health. Qualtrics is an online survey delivery service commonly utilized by researchers to reach diverse populations, including populations that may be challenging to access [59]. Participants (ages 18 to 24) were recruited through an email invitation from Qualtrics. Respondents were eligible to participate if they identified as Black and were between 18 and 24 years old. The online survey took 20 to 30 min to complete. Informed consent was obtained from all participants included in the study. The Institutional Review Board at The Ohio State University reviewed and approved all study protocols, ensuring compliance with ethical standards for research involving human subjects.

Our sample included 362 self-identified Black male and female youth, ages 18 to 24 (*M* = 21, *SD* = 1.96). Participants identified as African American (81%), Afro-Latino (3%), Afro-Caribbean/West Indian (10%), and Continental African (6%). Twenty-eight percent of participants of the sample self-identified as male, 70% self-identified as female, and a combined 0.08% of the sample identified as a transwoman, transman, nonbinary, or other. Fifty-nine percent of the sample reported being enrolled in school, and 30% reported being employed full-time. Most of the sample (41%) reported having a high school diploma, and 32% reported having some college, an associate’s degree, or trade school. Thirty-four percent of participants reported an annual household income of US $19,000 or less, and 38% reported household incomes (family of four or more) of more than US $100,000 annually. The majority of participants lived in Ohio (28%), Illinois (20%), or Michigan (15%).

## Measures

### Dependent Variab﻿le﻿s

#### Past Year Suicide Ideation

Suicide ideation was measured using a single item that asked respondents to indicate whether they had considered ending their life in the previous 12 months. Scholars have used a single-item measure of suicidal ideation with Black youth, young adults, and adults [26, 59–61]. Only 11.3% of participants that endorse a single-item suicide attempt engage in behavior that would not meet the standard definition of a suicide attempt. Similarly, only 8.8% of individuals who endorse a single-item measure of suicidal ideation endorse thoughts that would not meet standard definitions of suicidal ideation. These are indicative of an acceptable validity of a single-item measure of suicidal ideation [62]. Response categories were 1 = *yes* and 0 = *no* [63].

#### Depression Symptoms

We used the Center for Epidemiological Studies Depression Scale (CESD- 10) to measure depression symptoms. The CESD- 10 assesses depression symptoms experienced in the past week. It has been validated among clinically depressed populations, the general population, and sexual minorities of color [64, 65]. Sample items include the following: “How many times in the past week did you feel as good as other people?” and “How many times in the past week did you have trouble keeping your mind on task?” Response options range from 0 (*rarely or never*) to 3 (*most or all of the time*). The CESD- 10 scores range from 0 to 30, with higher scores indicating more depression symptoms (Cronbach’s alpha = 0.81). Any score above 10 classified Black youth as depressed. However, individuals who had scores above 20 were classified as having moderate to severe depression symptoms [66].

### Mediating Variable

#### Social Connectedness

We utilized the Social Connectedness Scale to assess the extent to which participants felt connected to others in social settings [35]. This scale consists of eight items, and participants were asked to rate their agreement with each item on a 6-point Likert scale, ranging from 1 (*strongly disagree*) to 6 (*strongly agree*). Negative items were reverse coded to ensure consistency. For example, one of the questions included in the scale is “I feel so distant from people.” Total scores on the scale were calculated by summing the individual item responses, resulting in scores ranging from 0 to 48. Higher scores indicate a greater sense of social connectedness. The scale has demonstrated high reliability, with internal consistency (Cronbach’s alpha) exceeding 0.92, and validity [35].

### Independent Variable

#### Everyday Discrimination

Everyday discrimination was measured using the Everyday Discrimination Scale, a 9-item Likert scale that evaluates the frequency of discriminatory encounters in daily life, with options ranging from 1 (never) to 5 (very often) [67]. The scale provides insight into the possible sources of these experiences, including ancestry, gender, race, or religion. The current study exclusively examined everyday discrimination in the context of race. Respondents were asked questions like the following: “How often have you been treated with less courtesy than others?” and “How often have others acted as if they are superior to you?” To analyze the data, the average score from all items was calculated for each participant. A higher average score indicates more frequent experiences of discrimination. The scale is highly reliable, evidenced by a strong Cronbach’s alpha of 0.88.

### Data Analysis Plan

Data were analyzed using Mplus version 8.3 [68]. The everyday discrimination measures were entered into two separate models, and path analyses were used to determine whether everyday discrimination is associated with depressive symptoms and suicide ideation and to uncover whether social connectedness mediates the relationship between everyday discrimination and depression symptoms and suicide ideation.

For Model 1, we used the chi-square test, comparative fit index (CFI), root-mean-square error of approximation (RMSEA), and standardized root-mean-square residual (SRMR) which were employed to indicate the adequacy of the model fit because the outcome (depression symptoms) was continuous [69]. Generally, the values of CFI ≧ 0.95, RMSEA ≦ 0.08, and SRMR ≦ 0.08 were considered a satisfactory model fit [70].

For Model 2, the weighted least squares means and variance (WLSMV) adjusted estimator was used instead of maximum likelihood estimation, as this estimator is preferred when the dependent variable (suicide ideation) is categorical. We did not provide model fit indexes in the study as the model was just identified for Model 2 [71]. Alternatively, we included standardized beta coefficients and the *p*-values used to examine associations among study variables. Standardized path coefficients (*β*) are reported for the direct effects, and unstandardized coefficients (*B*) for indirect effects were estimated for both models.

## Results

Table 1 provides the demographic characteristics of the sample. Fifty-one percent of the sample reported suicide ideation. The average score for depression symptoms in this sample was 15.50 (*SD* = 5.63; range: 0–30). A score exceeding 10 is indicative of depression. Among the study sample, 88% (*n* = 319) of participants scored higher than 10. The mean score for social connectedness was 0.38 (*SD* = 0.68), while the mean score for everyday discrimination was 3.00 (*SD* = 1.27). Correlation results (see Table 2) showed that depression symptoms were positively associated with suicide ideation (*r* = 0.29, *p* < 0.001). Social connectedness was negatively associated with depression symptoms (*r* = − 0.33, *p* < 0.001).

**Table 1:** Demographic variables of study participants (*N* = 362)

Variable	*n*	Percent
Sex assigned at birth		
Male	108	29
Female	254	71
Education		
College, postgraduate, or professional	24	7
Currently in school	32	9
High school diploma	144	41
Less than high school	10	3
Some college, AA degree, or trade school	114	32
Some high school	30	8
Employment status		
Homemaker	15	4
Employed for wages full time	104	30
Employed for wages part-time	119	34
Not employed	74	21
Unable to work (disabled)	10	3
Self-employed	30	8
Annual income		
Up to US $19,000	115	34
US $20,000 to US $39,000	16	5
US $40,000 to US $74,999	2	1
US $75,000 to US $99,999	7	2
US $100,000 to US $124,999	117	35
US $125,000 to US $149,000	2	1
US $150,000 or more	6	2
Suicide ideation		
Yes	183	51
No	177	49

Age range = 18–24, mean = 21.22; standard deviation = 1.90; depression symptoms range = 0–30, mean = 15.49, standard deviation = 5.63

**Table 2:** Correlation of study variables (*N* = 362)

Variables	M (range)	SD				
Suicide ideation			1			
Depression symptoms	15.49 (0–30)	5.63	0.29***	1		
Social connectedness	2.32 (1–6)	1.68	− 0.40***	− 0.33***	1	
Everyday discrimination	3.00 (1–5)	1.27	0.24***	0.30***	− 0.37***	1

***p <.001

### Path Analysis

**Fig. 1:**
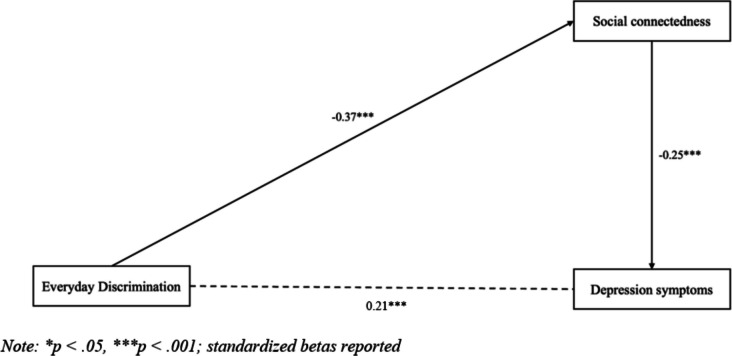
Path model testing direct and indirect effects from everyday discrimination to depression symptoms through social connectedness (*N* = 362)

The first path model, representing associations between everyday discrimination, social connectedness, and depression symptoms, is presented in Fig. 1. Discrimination was direct and negatively associated with social connectedness (*β* = − 0.37, *p* < 0.001). Social connectedness was direct and negatively associated with depression symptoms (*β* = − 0.25, *p* < 0.001) (see Table 3). Lastly, discrimination was indirect and positively associated with depression symptoms (*B* = 0.41, *p* < 0.001) (see Table 4).

**Table 3:** Direct effects on depression symptoms (*N* = 362)

Effect	*B*	*β*	*SE*	*p*-value	95% *CI*
Direct effects					
Social connectedness					
Everyday discrimination	− 0.20	− 0.37***	0.03	0.001	− 0.25, − 0.15
Depression symptoms					
Social connectedness	− 2.09	− 0.25***	0.44	0.001	− 2.95, − 1.23
Everyday discrimination	0.93	0.21***	0.23	0.001	0.47, 1.39

*p <.05, ***p <.001; *B*, unstandardized beta; *β*, standardized beta; *SE*, standard error; *CI*, confidence interval (for standardized betas)

**Table 4:** Indirect effects on depression symptoms (*N* = 362)

Effect	*B*	*SE*	*p-value*	95% *CI*
Everyday discrimination → Depression symptoms	0.41***	0.10	0.001	0.21, 0.62

***p <.001; *B*, unstandardized beta; *SE*, standard error; *CI*, confidence interval

**Fig. 2:**
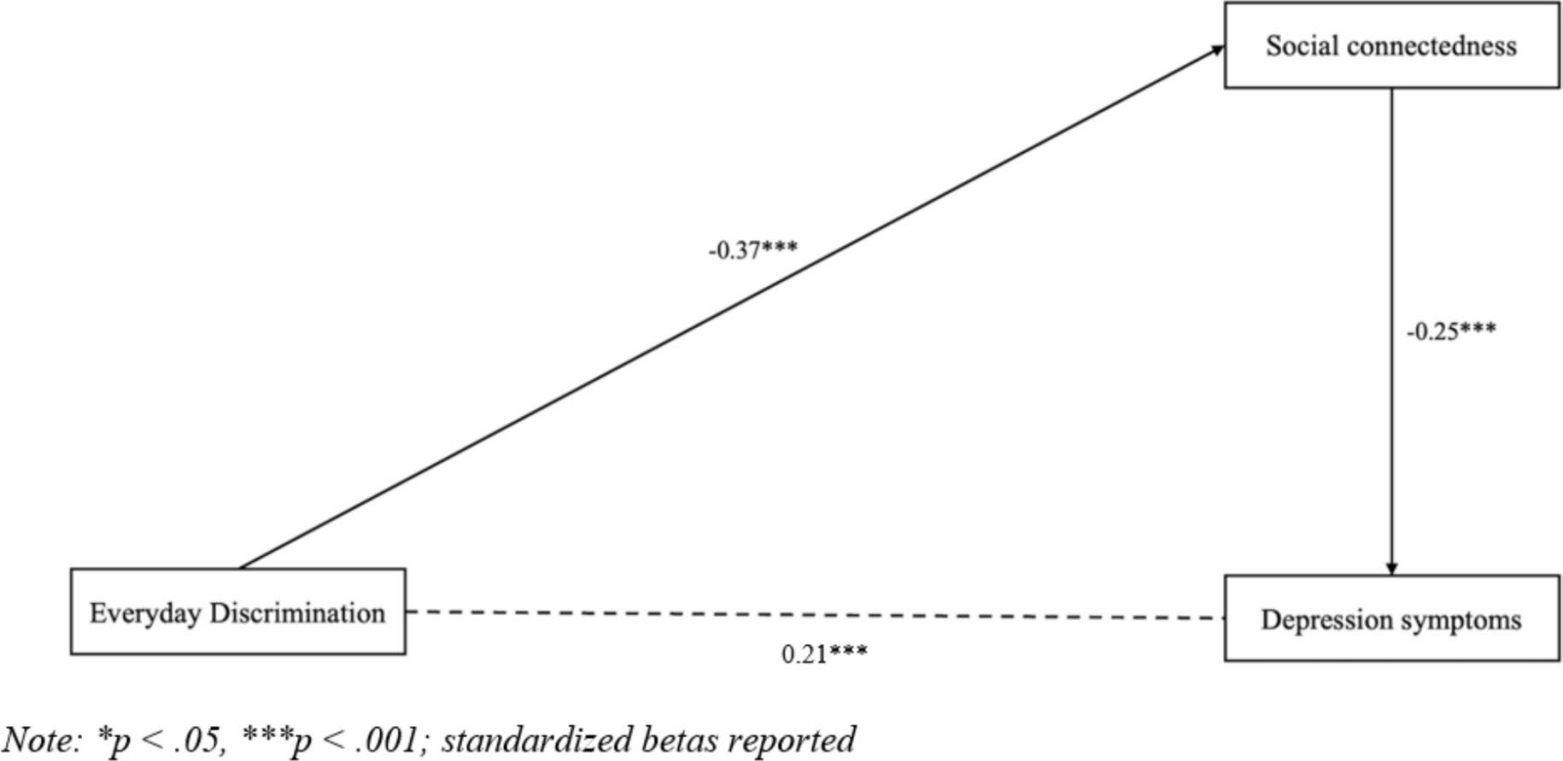
Path model testing direct and indirect effects from everyday discrimination to suicide ideation through social connectedness (*N* = 362)

The second path model, representing associations between discrimination, social connectedness, and suicide ideation, is presented in Fig. 2. Everyday discrimination was direct and negatively associated with social connectedness (*β* = − 0.37, *p* < 0.001). Social connectedness was direct and negatively associated with suicide ideation (*β* = − 0.35, *p* < 0.001) (see Table 5). Lastly, everyday discrimination was indirect and positively associated with suicide ideation (*B* = 0.05, *p* < 0.001) (see Table 6).

**Table 5:** Direct effects on suicide ideation (*N* = 362)

Effect	*B*	*β*	*SE*	*p*-value	95% *CI*
Direct effects					
Social connectedness					
Everyday discrimination	− 0.20	− 0.37***	0.03	0.001	− 0.25, − 0.15
Suicide ideation					
Social connectedness	− 0.26	− 0.35***	0.04	0.001	− 0.34, − 0.19
Everyday discrimination	0.04	0.10*	0.02	0.047	0.00, 0.08

*p <.05, ***p <.001; *B*, unstandardized beta; *β*, standardized beta; *SE*, standard error; *CI*, confidence interval (for standardized betas)

**Table 6:** Indirect effects on suicide ideation (*N* = 362)

Effect	*B*	*SE*	*p-*value	95% *CI*
Everyday discrimination → Suicide ideation	0.05***	0.01	0.001	0.03, 0.07

***p <.001; *B*, unstandardized beta; *SE*, standard error; *CI*, confidence interval

## Discussion

The purpose of the present study was to examine the associative relationships between discrimination and depression symptoms, and between discrimination and suicide ideation among Black youth, and whether social connectedness mediates the relationship between discrimination and behavioral health outcomes, including depression symptoms and suicide ideation. The results of the study indicated that discrimination was associated with both depression symptoms and suicide ideation.

Supporting the notion that social connectedness mediates the relationship between discrimination and depression symptoms, we found that discrimination had a direct and negative association with social connectedness, and social connectedness had a direct and negative association with depression symptoms. Similarly, supporting the idea that social connectedness mediates the relationship between discrimination and suicide ideation, we found that discrimination had a direct and negative association with social connectedness, and social connectedness had a direct and negative association with suicide ideation.

Examining social connectedness as a mediating variable, the current study indicates that social connectedness mitigated the impacts of discrimination on depression symptoms and suicide ideation. In other words, reports of social connectedness were associated with less reports of depression symptoms and suicide ideation. These findings are consistent with previous literature examining the impacts of social connectedness on reducing symptoms of depression and suicidality [38–40] and the significance of social connectedness in mitigating the impacts of discrimination on behavioral health outcomes [43]. In addition to this study’s consistency with previous literature, indicating the significance of social support as a protective factor against poor behavioral health outcomes, the present study provides novel evidence on the significance of social connectedness, a higher-order construct of social support, as a protective factor of depression symptoms and suicidality associated with discrimination among Black youth, a population in which the relationship between these constructs has been understudied.

Theoretically informed by social support theory, this study provides empirical evidence that perceived social integration and experiences of supportive interpersonal relationships positively impact Black youth’s mental well-being. Throughout their development and emergence into adulthood, Black youth experience greater stress than their white counterparts [72]. In the context of racial stress, the results of the present study contribute to the evidence of protective mechanisms that positively impact internalizing symptoms influencing the well-being of Black youth.

The study also affirms the prevalence of depression and suicide ideations among Black youth. More than half of the sample of Black youth included in the present study reported suicide ideation. The high percentage of suicidal ideation in our sample may reflect unique stressors, including structural inequities and COVID- 19-related challenges. One study with 379 adult participants aged 18–70 highlighted the strong connection between mental health conditions like depression and suicidal thoughts and both the experience of COVID- 19 and post-COVID symptoms [73]. The study also found that in some cases, suicidal thoughts and behaviors associated with COVID- 19 may have occurred before the individual contracted the virus. This suggests a potential link between mental health struggles like depression and vulnerability to COVID- 19, or that people who experienced mental health challenges may have already been experiencing suicidal ideation before infection. Both scenarios offer some support for the high percentage of participants who reported suicidal thoughts and ideations. In addition, the average score of depression among Black youth in the sample (*M* = 14) classified participants as depressed — a cutoff score of 10 or higher signifies significant depressive symptoms [66]. In addition to the relationship between discrimination and both depression and suicide ideation, the present study also found that depression was significantly associated with suicide ideation — supporting existing research examining depression as a precursor of suicidality [74–76]. The heightened rates of suicide among Black youth may be linked to an increased vulnerability to depression and suicidal thoughts stemming from chronic psychosocial stress, including racial discrimination. It is crucial for researchers and practitioners to recognize the disparities in suicidality prevalence, co-occurring psychiatric conditions, and the profound impact of race-related experiences on the mental well-being of Black youth.

In the quest for evidence-based solutions to tackle psychosocial stressors and racialized encounters that contribute to behavioral health disparities in Black youth, these insights offer important considerations for interventions and clinical approaches. When designing culturally responsive best practices to address depression symptoms and suicidal ideation related to discrimination, researchers and practitioners should consider the significance of social connectedness within the social networks of Black youth. These implications underscore the urgent need for research and practice to prioritize a holistic understanding of the unique challenges faced by Black youth to develop effective and culturally sensitive interventions that can mitigate the impact of discrimination and promote mental well-being within this vulnerable population.

### Limitations

Despite the strengths of the current study, there are limitations that should be recognized. The cross-sectional research design of the current study did not allow researchers to imply causality. In addition, the current study sample is not nationally representative of Black youth. Study participants were recruited from three Midwestern US states. Due to geographic differences, which may impact racialized experiences of discrimination, the generalizability of these results is limited. In addition, approximately 70% of the study sample identified as female. Due to the disproportionate representation of males and females, this may also pose a threat to generalizability. Another limitation of the study is that 38% of youth reported being part of families with four or more members earning over US $100,000. This may limit the generalizability of the findings to other populations. The use of a single-item measurement may have impacted the researchers’ ability to capture variation within suicide ideation, limiting measurement validity. The main outcome was evaluated using a single-item measure. Although this is a common approach in studying suicidal ideation among Black youth, young adults, and adults [26, 59–61], studies need to be conducted with more comprehensive measures to provide results with higher validity [77]. Future studies should examine these associations using validated scales with strong psychometric properties to ensure reliable findings [62]. In extending research on the influences of social connectedness on mitigating the impacts of discrimination on depression symptoms and suicidality, future research may further examine the impact particular types of social connectedness (e.g., family, friends, educators, mentors, or peers) have on behavioral health outcomes. In addition, future research may examine the significance of social connectedness within Black youth’s racial/cultural communities.

## Conclusion

The findings of our study suggest that everyday experiences of discrimination were significantly associated with depression symptoms and suicide ideation among Black youth. In examining whether social connectedness mitigated the impacts of discrimination on depression symptoms and suicidality, it was found that social connectedness mediated both the relationships between discrimination and depression symptoms and between discrimination and suicide ideation, as social connectedness was negatively associated with depression symptoms and suicide ideation. These findings provide further evidence for the role of social support, in the form of social connectedness, as a stress buffer among Black youth in mitigating the impacts of racial psychosocial stress, such as discrimination, on behavioral health outcomes, including depression and suicidality.

## Data Availability

The data and materials for this study support our empirical claims and comply with field standards.
